# Selective root canal retreatment of a maxillary first molar: a case report with a 9-year follow up

**DOI:** 10.3389/fdmed.2024.1422390

**Published:** 2024-08-21

**Authors:** Olavo Guerreiro Viegas, João Miguel Marques Santos

**Affiliations:** ^1^ From Private Practice Limited to Endodontics, ENDO EN ZO, Amsterdam, The Netherlands; ^2^ Institute of Endodontics, Faculty of Medicine, University of Coimbra, Coimbra, Portugal

**Keywords:** Cone Beam Computed Tomography (CBCT), endodontic outcome, minimally invasive, non-surgical retreatment, periapical radiograph (PR), selective root canal retreatment

## Abstract

Traditional endodontic retreatments usually target the entire root canal system. In contrast, selective root canal retreatment presents a new, targeted alternative that offers a less invasive solution. However, its promising approach faces potential obstacles due to a lack of long-term data, which might affect its widespread acceptance. This case report adds to the existing body of literature by offering an in-depth analysis of a long-term outcome following selective non-surgical retreatment, thereby bridging an important gap in knowledge. A 59-year-old male presented with post-treatment apical periodontitis (PTAP) in a maxillary first molar. Initial clinical examination revealed the patient was symptomatic, and the tooth responded negative to thermal tests and positive to vertical percussion. Radiographic assessment identified a radiolucency confined to the mesiobuccal root. No radiolucencies or signs of inflammation were observed in the distobuccal and palatal roots. These findings led to the decision to selectively retreat the mesiobuccal root non-surgically. This targeted approach aimed at addressing the inflammation while preserving the integrity of unaffected areas. The patient received selective non-surgical retreatment on the mesiobuccal root. During a nine-year follow-up, the patient remained asymptomatic, as confirmed by clinical observation. Periapical radiograph and Cone Beam Computed Tomography (CBCT) scan demonstrated complete healing of the treated root. Importantly, the untreated roots showed no signs or symptoms of apical periodontitis. This underscores the efficacy of the targeted treatment and its successful resolution of the inflammation. This case report aimed to show the long-term effectiveness and minimally invasive nature of selective root canal retreatment to address PTAP. It focused on the method's capacity to preserve tooth structure with minimum intervention. The positive outcomes highlight the urgent need for more controlled studies. Such research would confirm the advantages of selective retreatment, with the goal of improving endodontic protocols and patient care.

## Introduction

Post-treatment apical periodontitis (PTAP) is a pathology linked to root canal-treated teeth, mainly caused by microbiological factors, especially bacteria persisting within the root canal system or occasionally in the periradicular tissues (1). Studies have shown that most post-treatment diseases stem from clinical procedures that fall short of adequate standards, leading to ineffective control of root canal infections (2, 3). Despite adhering to the highest standards and procedural protocols, root canal treatments can still fail in approximately 5 to 15% of cases (4). This failure is often due to microbial infections that remain hidden in anatomical areas such as isthmuses, lateral canals, fins, and dentinal tubules. These areas are typically unreachable by the instruments and irrigants used in initial endodontic treatments (5). Furthermore, PTAP frequently results from root canal areas overlooked during the initial treatment (6).

In recent decades, managing teeth with PTAP has involved conservative methods such as surgical or non-surgical endodontic retreatment, intentional replantation, amongst others. These approaches offer a high probability of restoring the health of the periradicular tissues and retaining the tooth in the oral cavity (7). However, choosing among these conservative treatments involves considering several factors, including cost and associated risks, which may deter their use (6, 8–10).

Selective root canal retreatment is an emerging alternative for managing PTAP, focusing on targeted endodontic procedures. Unlike a full root canal retreatment, which addresses the entire previously treated root canal system regardless of the affected roots, the selective approach targets only the specific part of the root canal system showing signs of periapical disease (11). This method offers several advantages, including the creation of a conservative access cavity that is directed towards the affected root(s). This approach helps preserve the structural integrity of the tooth and any existing indirect restorations. Moreover, it may reduce the risk of iatrogenic errors and offer a more cost-effective option for patients (12).

Research on selective root canal retreatment is limited. To date, only two publications have focused on this procedure: a case report by Nudera (11) and a retrospective clinical study by Brochado Martins et al (12). Consequently, the long-term outcomes of selective root canal retreatment remain largely unexplored in the scientific literature.

The goal of this case report is to present the long-term outcome achieved through selective non-surgical root canal retreatment of a maxillary first molar. The distinctive feature of this case is the 9-year follow-up period, a characteristic currently unique in the literature for this treatment approach. By providing this data, we aimed to expand the knowledge and understanding of selective root canal retreatment, underlining its potential for sustained favorable outcomes over an extended period.

## Case description

This case report has been written according to Case Report Guidelines (CARE) 2013 (13) (Supplementary File S1).

**Figure 1:**
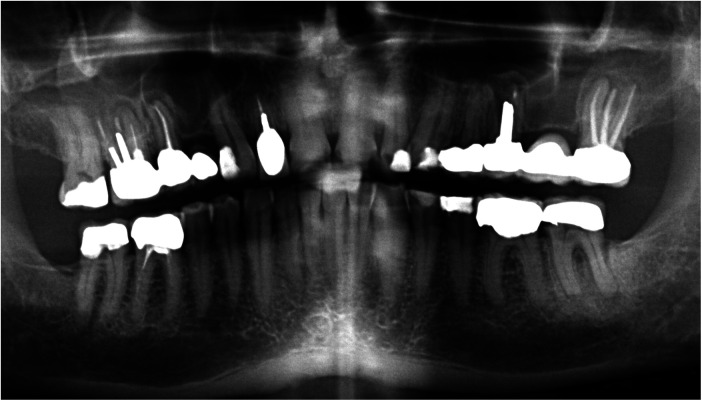
Orthopantomograph provided by the referring dentist at the time of patient referral to our clinic.

A 59-year-old Caucasian male, with no significant medical history, was referred to a private endodontic clinic. His dentist identified a periapical radiolucency in tooth 16 (FDI classification), which had undergone root canal treatment in the past. This tooth functioned as an abutment of a three-unit bridge, together with tooth 15 and a cantilever replacing tooth 14 (Figure 1). By the time of referral, tooth 15 had already been extracted and the bridge removed. The treatment plan, as outlined by the referring dentist, proposed using tooth 16 as an abutment for a new bridge. At the intake appointment, the patient reported experiencing minor discomfort on biting. Patient gave verbal, informed, and valid consent for the tooth examination. Clinical examination revealed that the tooth had a negative response to thermal tests, was not tender to palpation, vertical percussion test was positive, and mobility was physiological. A thorough examination of the gingival tissues surrounding the tooth indicated healthy conditions, evidenced by the absence of swelling and normal periodontal probing depths (all surfaces probed presented less than 3 mm depth). Furthermore, the tooth was restored with a composite filling on the buccal, occlusal, and mesial surfaces, with no signs of secondary caries.

A preoperative periapical radiograph was acquired using a digital radiography unit (XDR sensor, Cyber Medical Imaging, Los Angeles, CA, USA) with exposure parameters set at 70 kVp and 8 mA for 0.12 s, utilizing the paralleling technique (Rinn XCP-DS Fit Posterior Yellow, Dentsply Sirona, Konstanz, Germany) to ensure accurate imaging. The radiograph showed tooth 16 with previous root canal treatment, custom cast post and core extending into the middle third of the palatal and distobuccal canals. The periapical radiograph revealed a post extending into the beginning of the middle third of the distobuccal root, with a radiolucent area between the post and the gutta-percha. The gutta-percha appeared homogeneous and was positioned 0–2 mm from the radiographic apex. In the palatal root, a post extended into the beginning of the apical third, with a radiolucent area between the post and the gutta-percha, which appeared homogeneous and was positioned 0–3 mm from the radiographic apex. In the mesiobuccal root, a broken instrument was observed with no evident signs of a root filling, indicating possible untreated anatomy. Radiographic examination of the distobuccal and palatal roots' apices did not reveal any clear periapical pathology. CBCT imaging was not used before or during the treatment. The case was diagnosed as previously treated, symptomatic apical periodontitis of tooth 16. After reviewing the risks, benefits, and treatment options with the patient, verbal consent was obtained to perform a selective root canal retreatment of the mesiobuccal root on tooth 16. The tooth was anesthetized with 2% articaine with 1:100.000 adrenaline (Septodont, St Maur-des-Fosses, France). All procedures of the treatment were performed under an operating microscope (Carl Zeiss OPMI Pro Ergo, Oberkochen, Germany).

**Figure 2:**
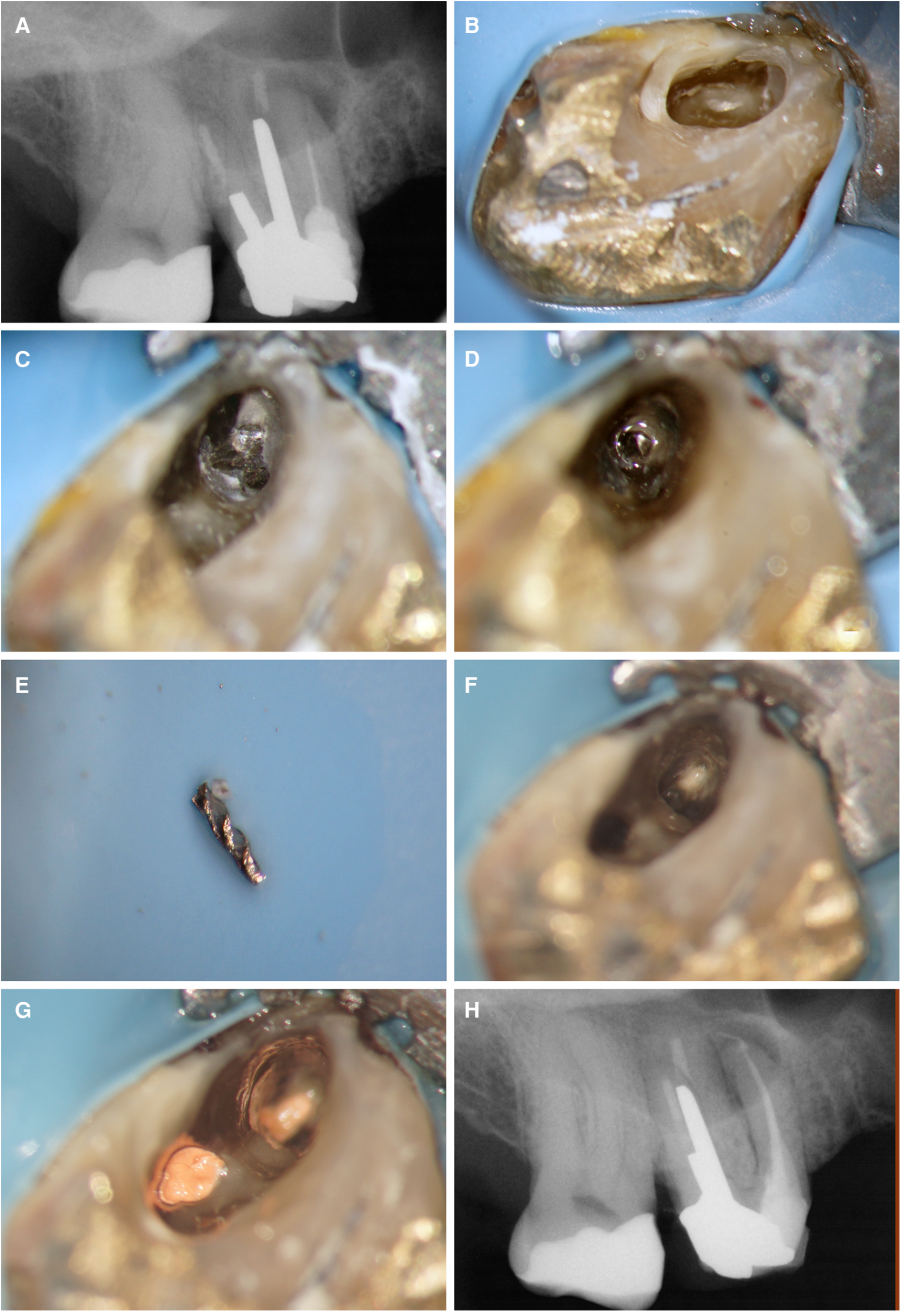
Overview of the endodontic procedure: **(A)** preoperative periapical radiograph, **(B)** access cavity preparation, **(C)** identification of the broken instrument within the canal, **(D)** creation of space surrounding the broken instrument, **(E)** successful removal of the broken instrument, **(F)** location and preparation of the MB2 canal, **(G)** root canal filling of both canals, and **(H)** postoperative periapical radiograph.

A conservative access cavity directed towards the root requiring retreatment was performed under rubber dam isolation, using a round end tapered diamond bur (TF-12SC, Mani Co., Tochigi, Japan) and copious water irrigation. The access was refined with ultrasonic tips (Start-X, Dentsply Maillefer, Ballaigues, Switzerland). The broken file was present in the mesiobuccal canal and was removed also by using ultrasonics (ET25, Satelec Acteon, Merignac, France). The untreated canal—mesiobuccal 2 - was identified. The working length was established electronically (Root ZX Mini, J. Morita Co., Kyoto, Japan). A patent reproducible glide path was created to size 20 using hand files (Ready•Steel FlexoFile, Dentsply Maillefer, Ballaigues, Switzerland) and enlarged to a size 25 with a rotary endodontic file (ProTaper Next® X2, Dentsply Maillefer, Ballaigues, Switzerland). Mesiobuccal 2 canal was merging with the mesiobuccal 1 canal in the middle third of the root. Irrigation protocol was performed using using a 30-G open-ended needle (NaviTip; Ultradent Products Inc, South Jordan, UT) attached to a 12-ml plastic syringe (Terumo Europe, Leuven, Belgium). The needle was inserted at 2 mm short of the working length. A total volume of 16 ml 6% NaOCl and 2 ml 17% EDTA (CanalPro™, Coltène Whaledent, Altstatten, Switzerland) were used per canal. As a final activation sequence, NaOCl was agitated ultrasonically (Irrisafe^™^; Satelec Acteon, Merignac, France). NaOCl was delivered 3 times using the same protocol described earlier. Ultrasonic activation was performed after each delivery for 3 periods of 20s each using size 20/.00 taper and a 21-mm Irrisafe file attached to an ultrasonic device on power setting 6 (P5 Newtron XS, Acteon Satelec). The canals were dried with paper points (ProTaper Next Absorbent Points X2, Dentsply Maillefer, Ballaigues, Switzerland). A master cone size X2 (ProTaper Next X2 Gutta-Percha Points, Dentsply Maillefer, Ballaigues, Switzerland) was selected and, in combination with an epoxy resin-based sealer, AH Plus (Dentsply International Inc, York, PA, USA), the root canal system was obturated using the continuous wave of condensation technique (System B Heat Source, Sybron Endo, Orange, USA and Obtura III Max System, Obtura Spartan, USA). The access was restored with a composite filling (SDR®, Dentsply Sirona, Konstanz, Germany and Filtek™ Z250 (3M, St. Paul, USA), and a final postoperative radiograph was captured (Figure 2). Postoperative instructions were given, and a follow-up appointment was scheduled in 6- and 12-months’ time. Patient was seen in the meanwhile for other root canal treatments, non-contributory for this case report, and reported no complaints or discomfort after the treatment.

The patient returned to the clinic 6 months later, symptom free. Clinical examination of tooth 16 revealed normal findings: the tooth had physiologic mobility, was not tender to palpation or percussion, and did not elicit any pain upon biting. The gingival tissues appeared healthy with no signs of swelling while exhibiting normal probing depths. A periapical radiograph of tooth 16 revealed a reduction in the size of the preoperative periapical radiolucency associated with the mesiobuccal root. At the time of the appointment, a 4-unit bridge spanning from tooth 13 to 16 had been previously placed by the referring dentist, demonstrating well-adapted margins at tooth 16. The patient reported no complaints, signs, or symptoms since the bridge was cemented and expressed satisfaction with the aesthetic outcome. Patient missed his 12 months follow-up appointment.

**Figure 3:**
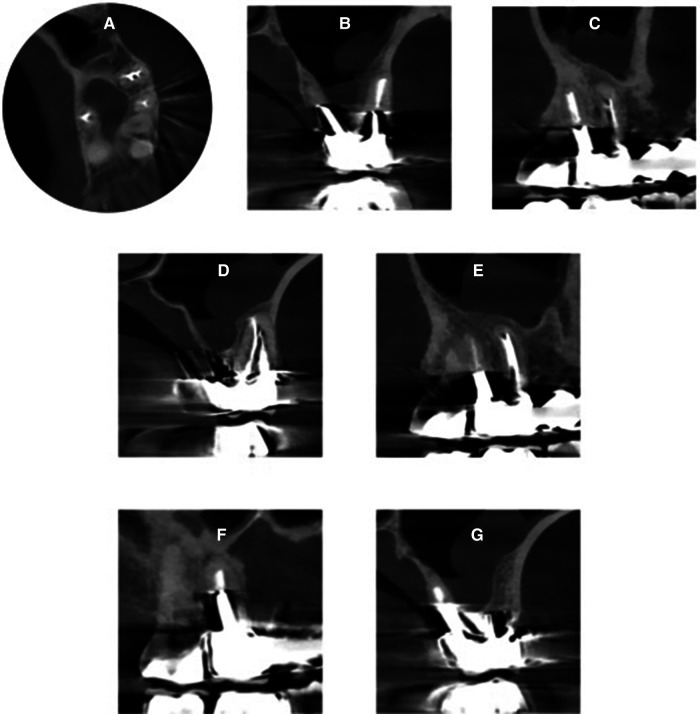
CBCT images taken 9 years post-initial treatment, distinctly illustrating the absence of periapical lesions around all roots: **(A)** axial view showing the middle third of tooth 16 and 17 (FDI classification), **(B)** coronal view showing the palatal and distobuccal roots of tooth 16, **(C)** sagittal view showing the palatal root of tooth 16, **(D)** coronal view showing the mesiobucal root of tooth 16, **(E)** sagittal view showing the mesiobuccal root of tooth 16, **(F)** sagittal view showing the palatal root of tooth 16, **(G)** coronal view showing the palatal root of tooth 16.

**Figure 4:**
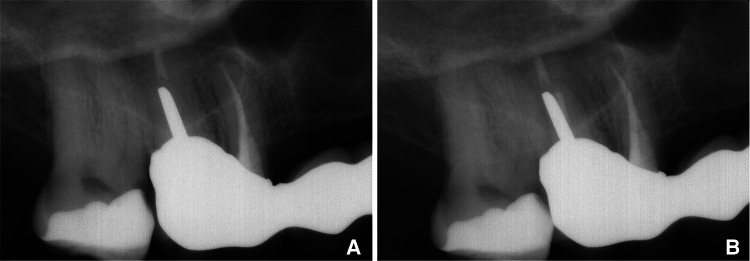
Comparative radiographs showcasing the 6-month follow-up post-treatment. **(A)** Alongside the 9-year follow-up **(B)**, Emphasizing the sustained treatment effectiveness and stability over time.

The patient was again referred to the private endodontic clinic, 9 years later, to assess another tooth. An intake appointment was planned and a CBCT (CS9600, Carestream Dental, LLC, Atlanta, GA) was done for diagnostic purposes. After the 2D and 3D scouting, the CBCT was taken with the following parameters: FoV 4 cm × 4 cm, high resolution, 75 mm voxel size, adult large, 0.7 mm Cu filter, 120 kV, 8 mA, with a scan time of 19 s. The tooth 16 was present in the same field of view and allowed us to assess the apical tissues. The CBCT scan showed complete healing of the periapical radiolucency associated with the mesiobuccal root and absence of a periapical lesion around the distobuccal and the palatal roots. Patient gave verbal consent to do a clinical examination and a periapical radiograph on the tooth 16. Clinical examination of tooth 16 revealed normal findings: the mobility was physiological, tooth was not tender to palpation or percussion, and did not elicit any pain upon biting. The gingival tissues appeared healthy with no signs of swelling while exhibiting normal probing depths. The periapical radiograph showed also complete healing. The examination of the tooth 16 and the periapical radiograph were done without any cost for the patient. The cost of the CBCT was declared with the examination of the tooth which the patient has been referred to assess (Figures 3, 4).

## Discussion

Endodontic retreatment traditionally follows an “all or none” approach, often leading to successful outcomes through full non-surgical methods. However, for teeth with recurring or persistent pathosis, this may not be the most conservative choice. The decision to opt for retreatment, extraction, or implant replacement varies among practitioners due to differing education backgrounds and personal values (7, 14).

Despite recent literature, selective retreatment is often overlooked as a management approach for apical periodontitis (7, 9). However, a new retrospective study suggests that less invasive strategies like selective retreatment could achieve similar success rates as conventional retreatment or apical surgery (7, 12, 15). This challenge established norms and signals a potential paradigm shift. The study's results, compared to those of conventional retreatment, underline the potential of selective retreatment as an equally effective, less invasive alternative. This comparison underscores the need for reconsidering our approach to managing apical periodontitis and the importance of presenting patients with this additional option during the decision-making process (10).

Current research on selective root canal retreatments is limited, primarily relying on data from a retrospective study that tracked outcomes over an average period of 15.6 months (12). This study underscores a significant gap in long-term outcome data and survival rates for this treatment approach, framing the current understanding of the long-term efficacy of selective retreatment.

Root-end surgery provides a unique perspective by adopting a selective approach that focuses solely on root(s) demonstrating periapical pathosis. This method departs from the traditional “all or none” strategy, with the aim of preserving healthy structures. Despite the excellent prognosis of apical surgery, there's an inherent risk of developing new disease manifestations in untreated roots (16). A study by Kraus et al. found that 8.1% of untreated roots in lower molars showed signs of periapical pathology five years post-apical surgery, as assessed by periapical radiographs (16). Despite the potential downside of untreated roots developing a lesion, it's crucial to acknowledge that the surgical approach aims only at roots exhibiting pathosis. This begs the question: why is retreatment viewed differently from apical surgery?

Technologies such as CBCT have stirred debate on their role in endodontic retreatment, with no consensus on their routine use for diagnosis and outcome assessment. However, research suggests that CBCT provides superior sensitivity and accuracy in detecting changes in periapical tissues. For instance, Davies et al. found that CBCT identified 27% more radiolucencies than conventional periapical radiographs (17), and Patel et al. demonstrated CBCT's superiority in identifying periapical radiolucencies in 19%–39% of cases, compared to traditional periapical radiographies (18).

Considering that selective retreatment primarily targets posterior and multi-rooted teeth, the value of CBCT is significant. These complex structures, especially molars, can pose challenges in lesion detection (6) and healing status evaluation using conventional periapical radiographs (17). Thanks to its advanced 3D imaging capabilities, CBCT provides a more detailed visualization (19, 20), enhancing not only the detection of apical periodontitis but also enabling a more accurate assessment of lesion healing. This makes it a valuable tool for increasing the accuracy and effectiveness of selective retreatment strategies.

Nudera proposed that selective root canal retreatment might only become accepted if CBCT is used for diagnosis (11). While there isn't enough evidence to definitively support or refute this claim, we can agree that relying solely on periapical radiographs might result in missed periapical lesions on root-filled teeth (6, 17, 19). Additionally, using only periapical radiographs makes it challenging to attribute a periapical lesion to a single root(s), a requirement for suggesting this treatment option to patients.

In contrast, a cadaver study by Kruse et al. found that 25%–50% of roots showing radiolucency in CBCT did not exhibit any periapical disease (21). This study should be interpreted with caution due to the lack of data on when the previous treatment was performed, which could potentially mask decreasing radiolucencies after root canal treatment. The Praxis Concept, which views periapical health and disease on a continuum, is crucial in understanding this study (14). Therefore, Kruse et al. study (21) might be better understood as a cross-sectional study that doesn't consider the progression of time and healing dynamics. The role of CBCT in diagnosis and outcome evaluation is a topic of active debate.

Reflecting on this case from 9 years ago, it's crucial to acknowledge the significant evolution in clinical decision-making. Initially, a CBCT scan was not conducted for the treatment. However, with the progression of knowledge and technology, the authors perspective has changed. Although the need to perform CBCT to confirm the diagnosis of the clinical case presented here remains debatable, recent evidence show that the additional information obtained from CBCT can influence the professional's confidence in establishing a predictable treatment planning (22). Currently, there is a strong acceptance that a CBCT scan is essential for precise evaluation of periapical conditions, especially in posterior teeth. In this specific case, obtaining a CBCT scan nine years ago would have provided us with more valuable information related to tooth 16.

Follow-up is essential in endodontics. While traditional focus has been on technical success, patient satisfaction is equally important. The patient expressed high satisfaction with the selective root canal retreatment, particularly appreciating the reduced duration of the procedure since only one root required attention. This not only alleviated discomfort but also lessened the financial burden. Additionally, the patient was reassured by the dentist's explanation that the conservative approach of removing less tooth structure would likely improve the tooth's longevity (23). Overall, the patient perceived these aspects as major benefits, enhancing comfort and confidence in the success of the treatment.

From a procedural standpoint, selective retreatment adopts a conservative approach, targeting only the specific root(s) in need of intervention. This targeted technique preserves more tooth structure compared to traditional methods, a benefit that, while empirically observed, still lacks robust scientific validation. Nevertheless, the preservation of tooth structure is associated with improved tooth survival rates (23, 24).

Selective retreatments are also performed through existing dental crowns, a method often preferred by patients as it avoids the need to remove the crown. While this approach is appealing, it carries the risk of crown damage, including potential microfractures in ceramic restorations, which could compromise the integrity and longevity of the crown (25). On the other hand, other studies indicate that conservative endodontic access in ceramic restorations does not significantly affect their fracture resistance (26). Selective retreatments are generally faster to perform than conventional retreatments, which reduces the chair time for patients. However, the major disadvantage of selective retreatment is the potential development of apical periodontitis in the untreated roots, a risk that is comparable to that of apical surgery.

Duncan et al. emphasized tooth survival as the main outcome for patients (27), along with evidence of periapical lesion reduction and normal periodontal ligament. This underscores the need for patient-centered outcomes and reveals patient concerns about reducing or eliminating periapical radiolucency. Using only periapical radiographs for diagnosis, presenting treatment options, and evaluating endodontic outcomes may not meet the expectations of both dentists and patients. If apical periodontitis poses significant local and systemic risks, then minimalist patient-focused outcomes like tooth survival may not be adequate (28).

Endodontic retreatment stands at a crossroads as traditional perspectives are reevaluated in the light of new evidence and viewpoints. The “all or none” approach could be replaced by more conservative strategies like selective retreatment. This new approach may provide opportunities to integrate patient preferences into the decision-making process and promote ethical dental care (10). Interestingly, when presented with various treatment options, patients typically opt for less invasive strategies than physicians (29). These ongoing debates and investigations are crucial in expanding the scope of endodontic retreatment and improving patient outcomes.

## Conclusion

Selective root canal retreatment, as an alternative to full retreatment, marks an innovative shift in endodontics. Despite limited literature on long-term outcomes, case studies like ours offer promising insights into its efficacy. However, the potential for new disease in untreated roots is a recognized risk, warranting careful consideration. As our understanding of these interventions matures, future high-quality clinical trials with larger sample sizes and extended follow-up periods are necessary to confirm these initial findings. Such research will provide crucial insights into the precise clinical situations where selective root canal retreatment may be most beneficial. As endodontics continues to evolve, it is incumbent upon us to prioritize strategies that optimize patient outcomes and advance the field.

## Data Availability

The original contributions presented in the study are included in the article/Supplementary Material, further inquiries can be directed to the corresponding author.

## Supplementary material

Supplementary File 1 Timeline of events describing the flowchart of the case according to case report guidelines.
