# Supplementary figures and images for: Selective root canal retreatment of a maxillary first molar: a case report with a 9-year follow up
Source: Front Dent Med. 2024 Aug 21;5:1422390. doi: 10.3389/fdmed.2024.1422390 (PMC11797766; doi:10.3389/fdmed.2024.1422390)

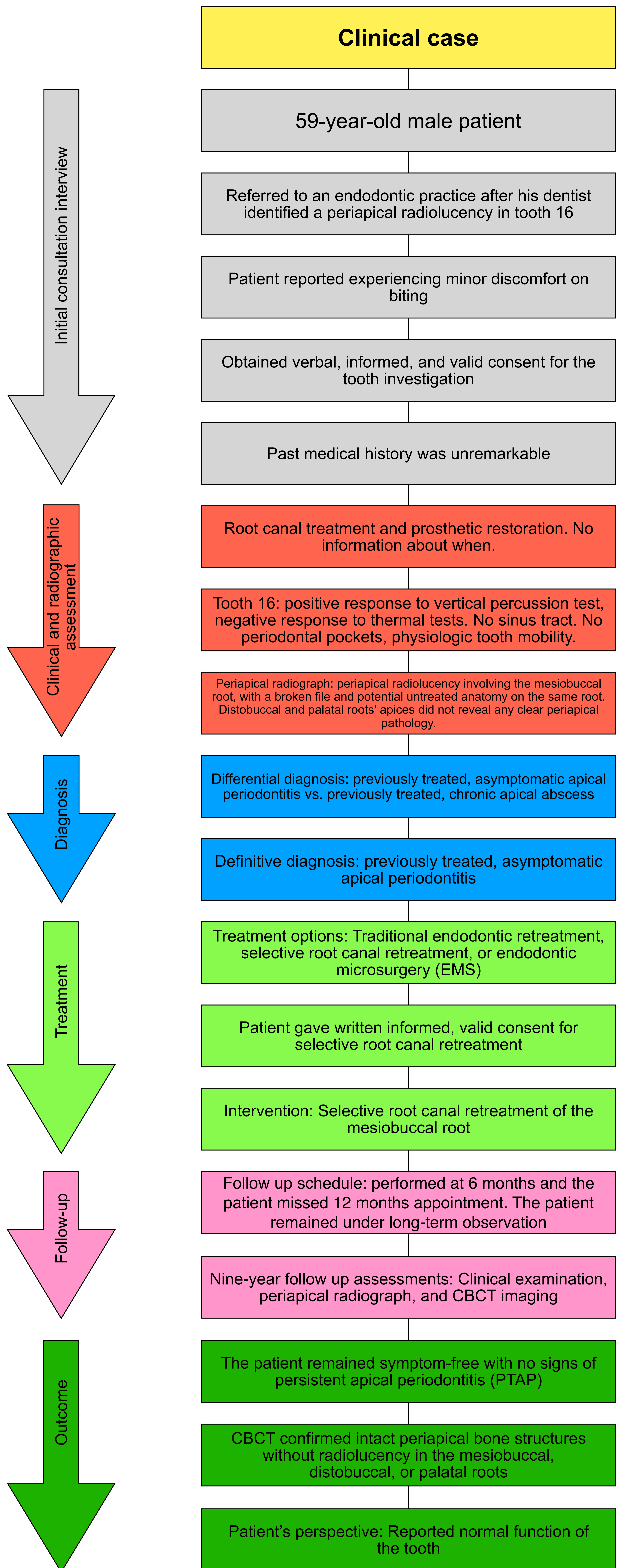

Supplement: Supplementary File 1 [file Datasheet1.pdf]
